# Self-monitoring of blood glucose in association with glycemic control in newly diagnosed non-insulin-treated diabetes patients: a retrospective cohort study

**DOI:** 10.1038/s41598-021-81024-x

**Published:** 2021-01-13

**Authors:** Hon-Ke Sia, Chew-Teng Kor, Shih-Te Tu, Pei-Yung Liao, Jiun-Yi Wang

**Affiliations:** 1 Division of Endocrinology and Metabolism, Department of Internal Medicine, Changhua Christian Hospital, 135 Nan-Hsiao Street, Changhua City, 500 Taiwan; 2 Department of Healthcare Administration, Asia University, 500 Liufeng Rd., Wufeng, Taichung City, 41354 Taiwan; 3 Internal Medicine Research Center, Changhua Christian Hospital, 135 Nan-Hsiao Street, Changhua City, 500 Taiwan; 4 Department of Medical Research, China Medical University Hospital, China Medical University, 91 Hsueh-Shih Rd., Taichung, 40202 Taiwan

**Keywords:** Diseases, Endocrinology, Health care, Medical research

## Abstract

The benefits of self-monitoring of blood glucose (SMBG) on glycemic control among type 2 diabetes (T2DM) patients not receiving insulin remains controversial. This study aimed to examine the association between SMBG and glycemic control in these patients. This retrospective longitudinal study enrolled 4987 eligible patients from a medical center in Taiwan. Data were collected from electronic medical records at 0 (baseline), 3, 6, 9, and 12 (end-point) months after enrollment. Patients were assigned to the early SMBG group or to the non-user group depending on whether they performed SMBG at baseline. Differences in glycated hemoglobin (HbA1c) reduction between groups at each time-point were assessed using SMBG group-by-time interaction in generalized estimating equations models, which were established using backward elimination method for multivariate regression analysis. Subgroup analyses for patients using non-insulin and insulin secretagogues were performed additionally. The estimated maximal difference in HbA1c reduction between groups (early SMBG users vs. non-users) was 0.55% at 3 months. Subgroup analyses showed maximal differences of 0.61% and 0.52% at 3 months in the non-insulin and insulin secretagogues groups, respectively. SMBG group-by-time interaction was statistically significant at 3 months and lasted for 12 months. The finding suggests that performing SMBG at disease onset was positively associated with better glycemic control in newly diagnosed non-insulin-treated T2DM patients, regardless whether non-insulin secretagogues or insulin secretagogues were used.

## Introduction

Diabetes has remained one of the most consequential chronic diseases worldwide considering the acute and chronic complications associated with poor glycemic control. Studies have shown that early glycemic control may have an extended beneficial effect lasting for at least 10 years, which can reduce the risk of serious micro- and macro-vascular complications—the so-called legacy effect or metabolic memory^1,2^. Therefore, newly diagnosed patients with diabetes should optimize glycemic control as soon as possible in order to lower the future risk of diabetes related complications more effectively.

Self-monitoring of blood glucose (SMBG) can assist patients with diabetes to better understand their glycemic status and consequently to adopt appropriate actions to cope with hyper- or hypoglycemia. SMBG has been shown to improve glycemic control among patients with diabetes receiving insulin therapy^3–5^. However, the benefits of SMBG on glycemic control among those with type 2 diabetes (T2DM) not receiving insulin has remained inconclusive^5–8^. Therefore, the National Institute for Health and Care Excellence guideline has yet to recommend routine SMBG among patients with T2DM not on insulin unless specific reasons emerge^9^. By contrast, the International Diabetes Federation (IDF) guideline suggests that SMBG should be considered at the time of diagnosis for non-insulin-treated patients with T2DM as a part of their education to facilitate timely treatment^10^. Most of the previous studies on SMBG had included patients with various diabetes durations, with only a few focusing on patients newly diagnosed with T2DM. However, conclusions in these studies are still inconsistent, including in randomized controlled trials (RCTs) and observational studies^11–14^.

Newly diagnosed patients with T2DM are at a critical juncture for health behavior change. Whether SMBG should be recommended to all newly diagnosed non-insulin-treated patients with T2DM remains an important practical issue for physicians and patients. Therefore, more evidence from real-world data is warranted to support its clinical effect. Furthermore, previous studies on SMBG did not analyze the effects resulting from different classes of anti-diabetic medications. Two broad categories are of particular concern: insulin secretagogues (insulin-releasing medications) with a potential adverse effect of hypoglycemia and non-insulin secretagogues that rarely cause hypoglycemia.

The current study aimed to examine the association between SMBG and glycemic control in newly diagnosed non-insulin-treated patients with T2DM and subgroups of patients receiving non-insulin and insulin secretagogues.

## Methods

### Subjects

This retrospective cohort study was conducted at the Changhua Christian Hospital (CCH), Taiwan. A total of 24,473 patients with T2DM were screened for eligibility using registry data from the Diabetes Case Management Program (DCMP) at the CCH Diabetes Care Center between January 2002 and December 2017. The DCMP provides standardized comprehensive diabetes care including lifestyle assessment, physical examination, laboratory evaluation, and diabetes self-management (DSM) education (such as instruction on nutrition, diet, exercise, medication, SMBG, and problem-solving skills aimed at reducing related complications). All participants in the program received education during scheduled teaching sessions. Care is delivered by a coordinated multidisciplinary team, including physicians, and certified diabetes educators (registered nurses and dietitians). A detailed description of the program has been reported elsewhere^15^. Diagnosis of T2DM was based on the criteria established by the American Diabetes Association^16^, by satisfying one of the following: a fasting plasma glucose value ≥ 126 mg/dL, a 2-h plasma glucose ≥ 200 mg/dL during a 75-g oral glucose tolerance test, a random plasma glucose ≥ 200 mg/dL in a patient with classic symptoms of hyperglycemia, or a glycated hemoglobin (HbA1c) level of ≥ 6.5%.

**Figure 1:**
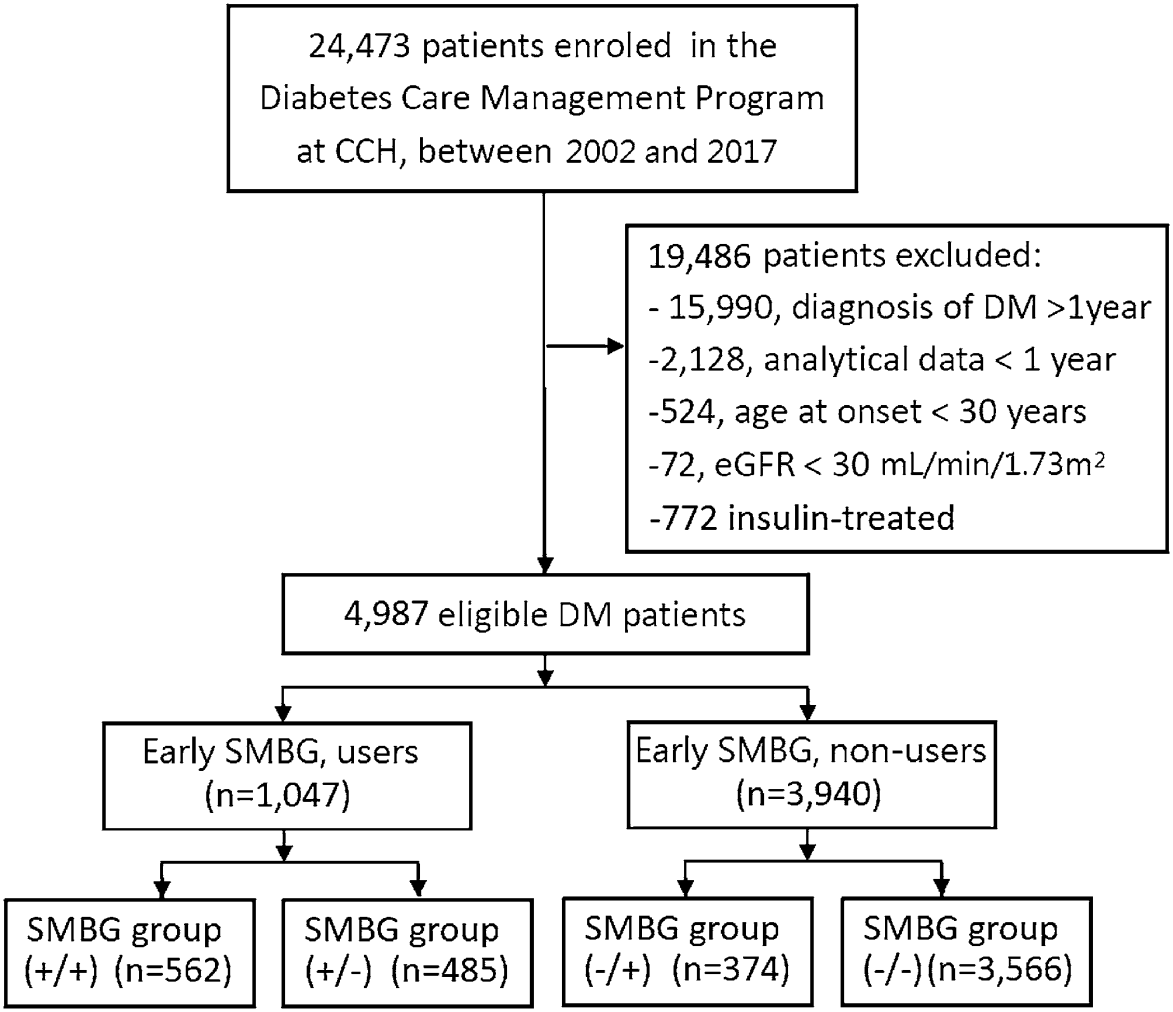
Flowchart of the study population. CCH, Changhua Christian Hospital; DM, diabetes mellitus; eGFR, estimated glomerular filtration rate; SMBG, self-monitoring of blood glucose.

Those receiving insulin (n = 727), those with < 1 year of analyzable data (n = 2128), those with diabetes duration longer than 12 months at the time of enrollment (n = 15,990), and those younger than 30 years of age (with greater likelihood of type 1 rather than type 2 diabetes) (n = 524) were excluded. Patients with an estimated glomerular filtration rate (eGFR) < 30 mL/min/1.73 m^2^ (n = 72) were also excluded given its undue effect on HbA1c levels and accuracy of glycemic status assessment^17^. Ultimately, 4987 eligible patients were identified for analysis (Fig. 1). Methods were performed in accordance with the relevant guidelines and regulations. The Institutional Review Board of CCH granted the waiver for informed consent and approved the study (IRB No: 191212).

### Data collection

Data were collected from the hospital’s electronic medical record systems, including the DCMP diabetes registry, prescriptions, laboratory data, and CCH research database. Diabetes specialists referred patients with T2DM to the Diabetes Care Center to participate in the DCMP, usually 2 to 6 weeks after the first outpatient clinic visit. After enrollment into the DCMP, all patients were filed in the basic data registry; underwent health-related behavior survey, physical examination, and laboratory testing; and attended all of the standardized one-on-one diabetes self-management (DSM) education sessions. After completing the course, a certified diabetes educator conducted face-to-face interviews and evaluated and recorded each patient’s knowledge regarding glycemic control, willingness toward DSM, frequency of performing SMBG, and medication adherence.

### Outcome variable: glycemic control

Glycemic control was assessed using HbA1c level, which was regarded as a continuous variable for analysis. HbA1c levels were measured upon enrollment into the DCMP (baseline values) and at 3, 6, 9, and 12 months thereafter. Serum HbA1c was measured through ion-exchange high-performance liquid chromatography using the VARIANTTM II Turbo system.

### Major exposure variable: self-monitoring of blood glucose

Frequency of performing SMBG was determined by checking the glucometers recordings and/or the patient’s (hand-written or electronic) diaries at baseline and at 12 months thereafter (end-point). SMBG was defined as self-assessment of blood glucose levels using a glucometer more than once per week. Participants were then categorized into early SMBG users and non-users groups based on availability of SMBG data at baseline. Considering that availability of baseline SMBG data does not represent continuation of SMBG throughout the entire year, data collected at the end-point was incorporated into analysis, after which participants were divided into four groups: SMBG group (+/+) (performed SMBG at baseline and end-point), SMBG group (−/−) (no SMBG at baseline and end-point), SMBG group (+/−) (SMBG at baseline but not at the end-point); and SMBG group (−/+) (SMBG at the end-point but not at baseline) (Fig. 1).

### Variables for subgroup analysis

Participants not receiving sulfonylureas or glinides during the observation period were categorized into the non-insulin secretagogue subgroup, whereas those receiving insulin secretagogues for ≥ 6 months were categorized into the insulin secretagogue subgroup. Subgroup analysis did not include those using insulin secretagogues for < 6 months. Insulin secretagogues included sulfonylureas and glinides, while non-insulin secretagogues included metformin, α-glucosidase inhibitor (acarbose), thiazolidinedione, and dipeptidyl peptidase 4 inhibitors. All anti-diabetic medications used for ≥ 1 month were collected for analysis.

### Other control variables

Basic data included age at onset of diabetes, gender, education level, and family history of diabetes. Health-related behaviors included smoking (within the preceding year), alcohol consumption (more than once weekly within the preceding year), and physical activity [regular (≥ 30 min/day, ≥ 3 days/week), occasional (less rigorous than regular exercise), or no exercise]. Knowledge regarding glycemic control was defined as an understanding of the need for and methods of controlling blood glucose. Willingness toward DSM was defined as the motivation to learn self-management techniques. Medication adherence was defined as taking medication regularly at the dose recommended by the physician over the past week. Four-point scales were used to assess the three aforementioned variables. Data were merged into simple dichotomies (i.e., top-two-box vs. bottom-two-box) and categorized as adequate (yes) or inadequate (no) for analysis.

Physical examination included measurement of blood pressure (BP), height, and body weight. Systolic and diastolic BP were measured with the patients in a seated position after a 10-min rest. Body mass index (BMI) was calculated as body weight (kg)/height (m^2^). Baseline laboratory data included total cholesterol (TC), high-density lipoprotein cholesterol (HDL-C), triglycerides (TG), low-density lipoprotein cholesterol (LDL-C), creatinine, and glutamic pyruvic transaminase (GPT) levels measured using a UniCel DxC 800 Synchron Clinical System (Beckman Coulter, Brea, CA, USA). eGFR was calculated using the equation recommended by the National Kidney Foundation^18^. Data on the 19 major non-psychiatric comorbidities described in the Charlson comorbidity index during the year preceding enrolment were collected from the CCH research database^19^. Major comorbidities, including congestive heart failure, coronary artery disease, and cerebrovascular accident, were analyzed as independent variables.

### Statistical analysis

Data were expressed as frequencies with percentages and means ± standard deviations (SD) for categorical and continuous variables, respectively. Differences among the four groups were assessed using the chi-square test for categorical variables and one-way analysis of variance for continuous variables. Generalized estimating equations (GEE) were used for the analysis of repeated HbA1c measurements (outcome variable). Statistical differences in HbA1c reduction between groups at each time-point were assessed using SMBG group-by-time interactions in GEE models established using the backward elimination method to select control variables. Subgroup analyses were performed to evaluate the association between SMBG and glycemic control in subgroups receiving different types of anti-diabetic medications. All analyses were two-tailed and conducted using IBM SPSS Statistics version 22 (IBM Corp., Armonk, NY, USA) with the significance level set at 0.05^20^.

## Results

### Characteristics of patients

A total of 4987 patients were identified (average age, 56.2 ± 11.5 years; 51.9% males). The SMBG group (+/+) was younger, had more males, had a higher education level, had more patients with a family history of diabetes, had better knowledge regarding glycemic control, had better willingness toward DSM, and was more physically active compared to the SMBG group (−/−) (Table 1). No significant differences were found in medication adherence, smoking, alcohol drinking, and BMI among the four groups.

**Table 1:** Basic characteristics of participants (n = 4987) in each SMBG group. Results are expressed as mean ± SD or n (%).

	SMBG group (+ / +), n = 562	SMBG group (+ /-), n = 485	SMBG group (-/ +), n = 374	SMBG group (-/-), n = 3566	*P value*
Age at onset (years)	54.4 ± 11.3	55.7 ± 12.1	53.5 ± 11	56.8 ± 11.5	< 0.001
Gender: male	322 (57.3%)	258 (53.2%)	218 (58.3%)	1789 (50.2%)	0.001
Level of education: no	26 (4.6%)	42 (8.7%)	17 (4.6%)	518 (14.5%)	< 0.001
Primary school	126 (22.4%)	146 (30.1%)	105 (28.1%)	1326 (37.2%)	
High school	261 (46.4%)	195 (40.2%)	168 (44.9%)	1248 (35.0%)	
University or above	149 (26.5%)	102 (21.0%)	84 (22.5%)	474 (13.3%)	
Family history of DM: yes	285 (50.7%)	252 (52.0%)	207 (55.4%)	1406 (39.4%)	< 0.001
Smoking	82 (14.6%)	66 (13.6%)	61 (16.3%)	566 (15.9%)	0.53
Alcohol drinking	43 (7.7%)	38 (7.8%)	23 (6.2%)	235 (6.6%)	0.58
Physical activity: no exercise	190 (33.9%)	219 (45.5%)	125 (33.4%)	2103 (59.7%)	< 0.001
Occasional exercise	136 (24.2%)	93 (19.3%)	94 (25.1%)	496 (14.1%)	
Regular exercise	235 (41.9%)	169 (35.1%)	155 (41.4%)	926 (26.3%)	
Knowledge regarding GC: yes	473 (91.7%)	392 (86.3%)	303 (83.2%)	1689 (51.7%)	< 0.001
Willingness toward DSM: yes	444 (86.1%)	404 (89.0%)	325 (89.3%)	2631 (80.5%)	< 0.001
Medication adherence: yes	498 (96.7%)	436 (96.3%)	351 (96.4%)	3162 (95.4%)	0.42
**Clinical variables**					
HbA1c at baseline (%)	8.8 ± 2.6	8.8 ± 2.5	9.0 ± 2.4	8.6 ± 2.5	0.006
BMI (kg/m^2^)	26.5 ± 4.5	26.5 ± 4.1	26.7 ± 4.4	26.5 ± 4.2	0.75
SBP (mmHg)	126.9 ± 16.5	128.8 ± 18.4	129.6 ± 16.5	132.3 ± 18.2	< 0.001
DBP (mmHg)	78.3 ± 10.3	78.0 ± 11.7	79.8 ± 11.1	80.1 ± 11.1	< 0.001
Total cholesterol (mg/dL)	170.8 ± 37.5	172.6 ± 36.9	179.5 ± 39.5	189.0 ± 43.1	< 0.001
Triglycerides (mg/dL)	139.5 ± 140.9	141.5 ± 94.9	144.2 ± 98.5	162.9 ± 153.3	< 0.001
HDL-C (mg/dL)	44.8 ± 11.7	45.4 ± 11.8	45.5 ± 11.7	47.9 ± 14.5	< 0.001
LDL-C (mg/dL)	99.6 ± 32.0	101.1 ± 30.8	107.3 ± 32.7	111.6 ± 34.2	< 0.001
eGFR (mL/min/1.73 m^2^)	99.3 ± 30.5	99.4 ± 32.7	99.1 ± 30.6	87.9 ± 28.0	< 0.001
GPT (U/L)	31.7 ± 29.0	30.3 ± 22.9	36.3 ± 31.7	34.3 ± 28.6	0.002
**Anti-diabetic medication**					
Insulin secretagogues	240 (42.7%)	222 (45.8%)	188 (50.3%)	2033 (57.0%)	< 0.001
Non-insulin secretagogues	258 (45.9%)	205 (42.3%)	143 (38.2%)	1191 (66.3%)	< 0.001

SMBG, self-monitoring of blood glucose; SD, standard deviation; DM, diabetes mellitus; GC, glycemic control; DSM, diabetes self-management; HbA1c, hemoglobin A1c; BMI, body mass index; SBP, systolic blood pressure; DBP, diastolic blood pressure; HDL-C, high-density lipoprotein cholesterol; LDL-C, low-density lipoprotein cholesterol; eGFR, estimated glomerular filtration rate; GPT, glutamic pyruvic transaminase.

The SMBG group (+/+) had higher baseline HbA1c levels and Charlson comorbidity index, and lower BP, TC, TG, LDL-C, and GPT levels than the SMBG group (−/−). During the observation period, the SMBG group (−/−) had more patients who needed insulin secretagogues (≥ 6 months) for glycemic control than the SMBG group (+ / +) (57.0% vs. 42.7%; p < 0.001). Moreover, the SMBG group (+/+) had more patients who used non-insulin secretagogues compared to the SMBG group (−/−), the lowest among the four groups (45.9% vs. 33.4%; p < 0.001).

### Association between SMBG and changes in HbA1c

Mean HbA1c reduction (unadjusted) from baseline to the end-point was 2.4% (95% confidence interval [CI], 2.2 to 2.6) in the SMBG group (+/+), 2.4% (95% CI, 2.1 to 2.6) in the SMBG group (−/+), 2.1% (95% CI, 1.9 to 2.4) in the SMBG group (+/−), and 1.7% (95% CI, 1.6 to 1.8) in the SMBG group (−/−) (Table 2). To determine the effect of early SMBG use, SMBG groups (+/+) and (+/−) were merged to form the “early SMBG users” group, while SMBG groups (−/−) and (−/+) were merged to form the “early SMBG non-users” group. Table 3 shows the difference in HbA1c reduction between both groups at each time-point using GEE to adjust for significant baseline characteristics, including age, gender, education level, smoking, baseline HbA1c, BMI, blood lipids, SBP, Charlson comorbidity index, medication adherence, physical activity, and anti-diabetic medication use. Variables included in the models were selected using the backward elimination method.

**Table 2:** Mean HbA1c levels during the observation period, anti-diabetic medications, and main comorbidities in each SMBG group. Results are expressed as mean ± SD, difference in mean (95% CI), or n (%).

	SMBG Group (+ / +), n = 562	SMBG Group (+ /-), n = 485	SMBG Group (-/ +), n = 374	SMBG Group (-/-), n = 3566	*P-value*
**SMBG frequency (per week)**					
At baseline	5.9 ± 5.6	5.0 ± 5.3			
At 12 months	3.2 ± 2.7		2.9 ± 2.7		
**HbA1c (%)**					
At baseline	8.8 ± 2.6	8.8 ± 2.5	9.0 ± 2.4	8.6 ± 2.5	0.006
At 3 months	6.5 ± 0.9	6.6 ± 0.9	6.8 ± 1.2	7.0 ± 1.4	< 0.001
At 6 months	6.4 ± 0.8	6.6 ± 0.9	6.6 ± 1.1	6.8 ± 1.2	< 0.001
At 9 months	6.4 ± 0.8	6.7 ± 1.0	6.7 ± 1.1	6.9 ± 1.2	< 0.001
At 12 months	6.4 ± 0.8	6.7 ± 1.0	6.6 ± 1.1	6.9 ± 1.2	< 0.001
Difference in mean between baseline and end-point	2.4 (2.2 to 2.6)	2.1 (1.9 to 2.4)	2.4 (2.1 to 2.6)	1.7 (1.6 to 1.8)	
**Insulin secretagogues**					
Sulfonylurea	253 (45.0%)	226 (46.6%)	177 (47.3%)	1797 (50.4%)	0.050
Glinides	54 (9.6%)	70 (14.4%)	56 (15.0%)	571 (16.0%)	0.001
**Non-insulin secretagogues**					
Metformin	522 (92.9%)	447 (92.2%)	326 (87.2%)	2755 (77.3%)	< 0.001
DPP-4 inhibitors	123 (21.9%)	104 (21.4%)	68 (18.2%)	277 (7.8%)	< 0.001
Thiazolidinediones	30 (5.3%)	25 (5.2%)	18 (4.8%)	289 (8.1%)	0.004
Acarbose	80 (14.2%)	64 (13.2%)	59 (15.8%)	519 (14.6%)	0.754
Comorbidity: CCI	2.0 ± 1.4	1.9 ± 1.3	1.8 ± 1.2	1.8 ± 1.2	0.003
CHF	88 (15.7%)	75 (15.5%)	44 (11.8%)	373 (10.5%)	< 0.001
CAD	36 (6.4%)	26 (5.4%)	28 (7.5%)	295 (8.3%)	0.082
CVA	43 (7.7%)	30 (6.2%)	11 (2.9%)	228 (6.4%)	0.029
Cancer	26 (4.6%)	15 (3.1%)	13 (3.5%)	70 (2.0%)	0.001

SMBG, self-monitoring of blood glucose; SD, standard deviation; CI, confidence interval; HbA1c, hemoglobin A1c; DM, diabetes mellitus; DPP-4, dipeptidyl deptidase 4; CHF, congestive heart failure; CAD, coronary artery disease; CVA, cerebrovascular accident.

**Table 3:** Longitudinal HbA1c trajectory by generalized estimating equations. The models were adjusted for age at onset, gender, level of education, smoking status, physical activity, medication adherence, HbA1c at baseline, body mass index, systolic blood pressure, triglycerides, high-density lipoprotein cholesterol, low-density lipoprotein cholesterol, using insulin secretagogues, and Charson comorbidity index. Results are expressed as regression coefficients (β) with their corresponding standard error (se). Backward elimination method was adopted to select variables. Abbreviations: SMBG, self-monitoring of blood glucose; HbA1c, hemoglobin A1c.

	Early SMBG		1-year SMBG	
	Group (+ / + , + /-) vs Group (-/-, -/ +)		Group (+ / +) vs Group (-/-)	
	Adjusted β (se)	P-value	Adjusted β (se)	P-value
**SMBG group:** no				
Yes	0.21 (0.06)	0.001	0.23 (0.08)	0.007
**Time: baseline**				
3 months	− 1.69 (0.04)	< 0.001	− 1.64 (0.04)	< 0.001
6 months	− 1.84 (0.04)	< 0.001	− 1.79 (0.04)	< 0.001
9 months	− 1.80 (0.04)	< 0.001	− 1.75 (0.04)	< 0.001
12 months	− 1.79 (0.04)	< 0.001	− 1.74 (0.04)	< 0.001
**Interaction of SMBG group and time**				
3 months	− 0.55 (0.09)	< 0.001	− 0.64 (0.12)	< 0.001
6 months	− 0.45 (0.09)	< 0.001	− 0.57 (0.12)	< 0.001
9 months	− 0.47 (0.09)	< 0.001	− 0.63 (0.12)	< 0.001
12 months	− 0.45 (0.09)	< 0.001	− 0.60 (0.12)	< 0.001

**Figure 2:**
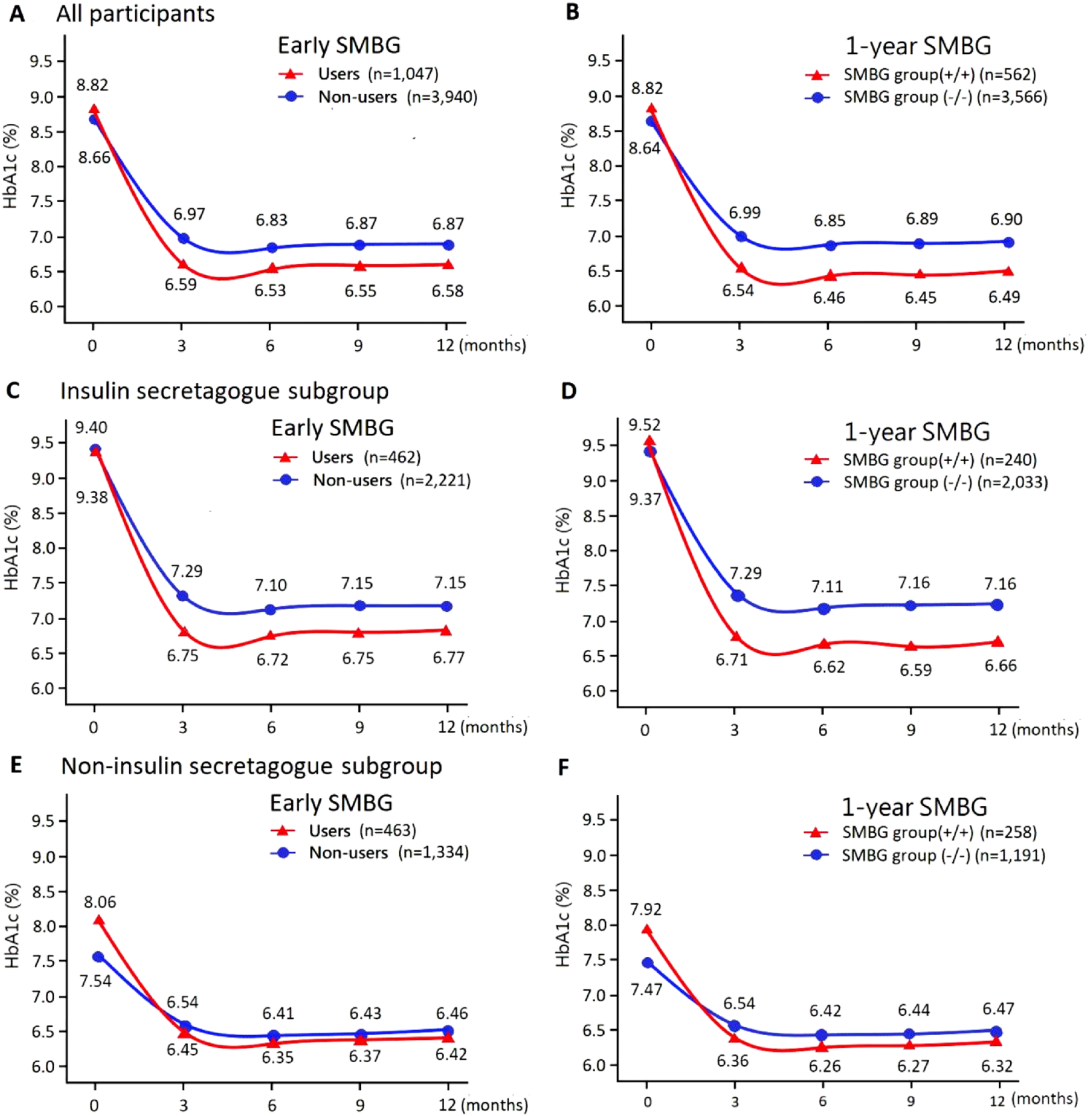
Model-based mean HbA1c values and longitudinal HbA1c trajectory after adjustment of confounding variables by generalized estimating equations: (**A**) Early SMBG, users: SMBG group (+/+,+/–) versus non-users: SMBG group (–/+, –/–), in all participants; (**B**) 1-years SMBG, SMBG group (+/+) versus SMBG group (–/–), in all participants; (**C**) Early SMBG, users versus non-users, in insulin secretagogues subgroup; (**D**) 1-years SMBG, SMBG group (+/+) versus SMBG group (–/–), in insulin secretagogues subgroup; (**E**) early SMBG, users vesus non-users, in non-insulin secretagogues subgroup; (**F**) 1-years SMBG, SMBG group (+/+) versus SMBG group (–/–), in non-insulin secretagogues subgroup. All the p-values of interaction terms of SMBG group and time were ≤ 0.002. Details are presented in Supplementary Table S1. SMBG, self-monitoring of blood glucose; HbA1c, hemoglobin A1c.

Model-based estimated mean HbA1c values and longitudinal HbA1c trajectory after adjustment of other control variables are shown in Fig. 2. Accordingly, both groups showed a decrease in HbA1c during the observation period. Early SMBG users had a lower estimated HbA1c level than early SMBG non-users, with the maximal difference being 0.55% at 3 months and minimum difference being 0.45% at 6 and 12 months (Fig. 2A & Supplementary Table S1). A comparison between SMBG groups (+/+) and (−/−) showed an even greater difference in HbA1c reduction, with the maximal difference being 0.64% at 3 months and minimum difference being 0.57% at 6 months (Fig. 2B & Supplementary Table S1).

### Subgroup analysis

In the insulin secretagogue subgroup, early SMBG users and non-users had similar baseline estimated HbA1c levels (9.38% vs. 9.40%) (Fig. 2C & Supplementary Table S1). Patients who performed early SMBG achieved greater HbA1c reduction than those who did not, with the maximal difference being 0.52% at 3 months and the minimum difference being 0.36% at 6 and 12 months. A comparison between SMBG groups (+/+) and (−/−) showed an even greater difference, with the maximal difference being 0.72% at 3 and 9 months and the minimum difference being 0.63% at 6 months (Fig. 2D & Supplementary Table S1). In the non-insulin secretagogue subgroup, early SMBG users had a much higher estimated baseline HbA1c than early SMBG non-users (8.06% vs. 7.54%). Nonetheless, the former had greater HbA1c reduction than the latter, with the maximal difference being 0.61% at 3 months and the minimum difference being 0.56% at 12 months (Fig. 2E & Supplementary Table S1). The SMBG group (+/+) showed even greater HbA1c reduction than the SMBG group (−/−), with the maximal difference being 0.63% at 3 months and the minimum difference being 0.60% at 12 months (Fig. 2F & Supplementary Table S1). Basic characteristics of participants in each subgroup are shown in Supplementary Tables S2 to S5. The details of GEE models in each subgroup are shown in Supplementary Tables S6 and S7.

To assess the association between frequency of SMBG and glycemic control, early SMBG users were further divided into the < 7 times/week group and the ≥ 7 times/week group according to frequencies of SMBG at baseline. The latter group had significantly greater decrements in HbA1c reduction which reached a difference of 1.27% or more, compared with early SMBG non-users (Supplementary Table S8).

## Discussion

The current study found that SMBG was associated with better glycemic control in newly diagnosed non-insulin-treated patients with T2DM in a clinical practice setting. The extra decrements in HbA1C reduction in early SMBG users compared with non-users were greater than 0.45% at all the time-points during the one-year follow-up. Most of the previous SMBG studies are RCTs that mainly included patients with various T2DM durations. Given the heterogenous baseline characteristics and SMBG-incorporated clinical interventions among such studies, the reported intervention effects of SMBG are inconsistent. Some systematic reviews and meta-analyses showed differences in HbA1c reduction between groups approximately ranging from 0.1% to 0.4%^21–25^.

Islet cell function, diabetes-related knowledge, attitudes, and self-management ability among newly diagnosed patients with diabetes differ from those who have experienced the disease longer. Some studies suggested that newly diagnosed patients with T2DM are new to SMBG and would benefits more from SMBG than prevalent users^10,26^. Nonetheless, others have suggested that newly diagnosed patients with T2DM might have improved glycemic control despite limited input from health care professionals^27^. Only a few studies have provided data regarding the effect of SMBG on glycemic control in newly diagnosed patients with T2DM. An RCT by O’kane et al. showed a decrease of mean HbA1c levels after 12 months from 8.8 (± 2.1 [SD]) to 6.9 (± 0.8)% in the SMBG group, and from 8.6 (± 2.3) to 6.9 (± 1.2)% in the control group^11^. There were no significant differences between groups in HbA1c at any time- point. Whereas another RCT found that SMBG users had significant greater reduction in median HbA1c levels, from 6.6 to 6.1% (p < 0.05), after 1 year of follow-up, but no change in the control group^12^.An observational study by Virdi et al. demonstrated a difference of 0.4% (p = 0.034) and 0.3% (p = 0.039) in HbA1c reduction between SMBG and non-SMBG patients with newly diagnosed T2DM at 12 and 36 months, respectively^13^.

The current study found that SMBG was associated with better glycemic control, with even greater differences in HbA1c reduction between SMBG users and non-users than previously reported. The discrepancy in the reported intervention effect may be explained as follows. First, the non-randomized nature of retrospective studies could not preclude the possibility that patients who perform SMBG tend to be better motivated. Therefore, patients performing SMBG may tend to have better glycemic control due to their personal characteristics. The current study found that patients who performed SMBG had a higher education level, had better knowledge regarding glycemic control, had better willingness toward DSM, and were more physically active than those who did not. To address this issue, multivariable models were used to adjust for confounding variables and therefore improve subject comparability and the validity of the results. In contrast to the observational study by Virdi et al., it utilized propensity score matching for analysis without adjusting for participants’ knowledge, attitude, and behavioral factors^13^. Second, while RCTs provide stronger support for causality, some inherent limitations exist in SMBG-related RCTs, such as the Hawthorne effect wherein patients in the control group may improve their behavior due to the attention from researchers^10^. This effect may be even more obvious among newly diagnosed patients with T2DM and thus attenuate SMBG-related intervention effects in RCTs^26^. On the other hand, observational studies provide valuable insight into glycemic outcome under conditions of routine patient care^10^. Third, the RCT by O’kane et al. used a rigorous treatment algorithm based on the HbA1c target^11^. Applying uniformly strict measures to the control group may directly improve their glycemic control and thus obscure the potential benefit of SMBG^8,10^.

Insulin secretagogues can stimulate β-cell secretion of insulin, with weight gain and hypoglycemia as potential side effects. To avoid such side effects, insulin secretagogues are usually reserved as a second choice for those with impaired β-cell function and poor glycemic control^28^. Given that careful dosage titration of insulin secretagogues are needed to safely achieve sustained glycemic control, those receiving such medication theoretically rely on SMBG more than those receiving non-insulin secretagogues. No study has yet analyzed the glycemic benefit of SMBG on patients receiving insulin and non-insulin secretagogues. However, some studies have stated that patients on non-insulin secretagogues are at very low risk for hypoglycemia and generally do not require SMBG^23^.

The subgroup analysis conducted herein demonstrated that SMBG was associated with significant lower HbA1c levels in patients using insulin secretagogues. Among patients using non-insulin secretagogues, the HbA1C levels achieved during follow-up were similar irrespective of SMBG use, which may be explained by the floor effect of glycemic control. Nonetheless, SMBG users had greater HbA1C reduction than SMBG non-users due to higher baseline HbA1C levels. This has been the first study to provide evidence supporting the association of SMBG use with favorable glycemic control among newly diagnosed patients with T2DM receiving non-insulin secretagogues. Notably, although SMBG users had a higher mean baseline HbA1c level, they were less frequently prescribed insulin secretagogues and showed an even lower mean HbA1c level at the end-point compared to SMBG non-users. This suggest that performing SMBG could reduce the probability of being prescribed insulin secretagogues, potentially reducing the risk for hypoglycemia. Our findings support the IDF guidelines recommending SMBG in newly diagnosed patients with T2DM receiving either non-insulin or insulin secretagogues.

A recent observational study showed that increased frequency of SMBG was associated with better glycemic control and more weight loss compared with less frequent SMBG among patients with T2DM and obesity irrespective of insulin use^4^. Another meta-analysis reported that performing SMBG 8 to 14 times weekly was more likely to have an improved glycemic control^25^. Our observation regarding the association of higher SMBG frequency (≥ 7 time/week) with greater HbA1c reduction was in line with these studies.

The current study has several limitations worth being noted. First, the inference of cause-and-effect relationship of this study was limited by the lack of randomization. Second, observational studies may suffer from reverse causality. For instance, patients with poor glycemic control tend to be prescribed SMBG, which may attenuate the SMBG intervention effect^26^. Our study did find some evidence of such a condition in the SMBG group (−/+). However, after incorporating follow-up data, HbA1c reduction in this group was even greater than that of the SMBG group (−/−), thus mostly eliminating this concern. Third, our grouping was based on SMBG data at baseline, which may not be representative of SMBG use or non-use throughout the whole year. Incorporating SMBG data at the end point can somewhat reduce information bias and misclassification of SMBG status. Forth, data on financial status (income), which might affect SMBG performance and glycemic control, were lacking. The DCMP has been funded by Taiwan National Health Insurance Bureau. However, the insurance does not reimburse blood glucose test strips for patients with T2DM. It is possible that financial reasons discouraged some patients from performing SMBG. Given that financial status is usually influenced by age, gender, and education^29^, controlling for such variables would be somewhat helpful to reduce potential biases. Fifth, patients performing SMBG may have better diet control and thus explain the greater HbA1c reduction. However, our study lacked dietary information for statistical adjustment. SMBG alone without any other actions is unable to reduce blood glucose and therefore it must be accompanied by diabetes education and treatment. Hence, isolating the effect of SMBG on glycemic control from other accompanying measures is difficult. The benefits of SMBG lie within its exposition of blood glucose values that subsequently assist patients and health care providers in understanding their glycemic status, through which correct strategies, such as medication adjustment, diet, and physical activities, can be adopted to safely achieve individual glycemic control goals^8,10,12^.

## Conclusion

This retrospective cohort study supports actively recommending SMBG for the treatment of newly diagnosed patients with T2DM from disease onset. Our results also showed that early SMBG use may be associated with favorable glycemic control, irrespective of using non-insulin or insulin secretagogues. The findings obtained herein can be complementary to those presented in RCTs and can provide additional evidence for reference in those with similar conditions.

## Supplementary information

Supplementary Information.

## Data Availability

The dataset used in the study is not available. Data are confidential according to Personal Information Protection Act implemented by the Taiwanese Government in 2012. Further information on data acquisition is available from the first author upon reasonable request.
