# Supplementary material for: Self-monitoring of blood glucose in association with glycemic control in newly diagnosed non-insulin-treated diabetes patients: a retrospective cohort study
Source: Sci Rep. 2021 Jan 13;11:1176. doi: 10.1038/s41598-021-81024-x (PMC7806592; doi:10.1038/s41598-021-81024-x)
Supplement: Supplementary file 1 — Supplementary Information. [file 41598_2021_81024_MOESM1_ESM.pdf]

Title of article:

Self-monitoring of blood glucose in association with glycemic control in newly diagnosed non-insulin-treated diabetes patients: a retrospective cohort study

Names of authors:

Hon-Ke Sia[1,2] , Chew-Teng Kor[3], Shih-Te Tu[1], Pei-Yung Liao[1], Jiun-Yi Wang[2,4,*]

Affiliated organizations:

[1] Division of Endocrinology and Metabolism, Department of Internal Medicine, Changhua Christian Hospital, 135 Nan-Hsiao Street, Changhua City 500, Taiwan

[2] Department of Healthcare Administration, Asia University, 500 Liufeng Rd., Wufeng, Taichung City 41354, Taiwan

[3] Internal Medicine Research Center, Changhua Christian Hospital, 135 Nan-Hsiao Street, Changhua City 500, Taiwan

[4] Department of Medical Research, China Medical University Hospital, China Medical University, 91 Hsueh-Shih Rd., Taichung 40202, Taiwan

[*]**Corresponding author:**

Jiun-Yi Wang, Ph.D.

500 Liufeng Rd., Wufeng, Taichung City 41354, Taiwan

e-mail: jjwang@asia.edu.tw

Supplementary Table S1

Estimates mean HbA1c levels and differences in HbA1c reduction at each time-point after adjustment of confounding variables by generalized estimating equations.

Time (months)	n	0	3	6	9	12
All participants						
Early SMBG	1047	8.82	6.59	6.53	6.55	6.58
No early SMBG	3940	8.66	6.97	6.83	6.87	6.87
Difference in HbA1c reduction			-0.55	-0.45	-0.47	-0.45
P-value			<0.001	<0.001	<0.001	<0.001
SMBG	562	8.82	6.54	6.46	6.45	6.49
No SMBG	3566	8.64	6.99	6.85	6.89	6.90
Difference in HbA1c reduction			-0.64	-0.57	-0.63	-0.60
P-value			<0.001	<0.001	<0.001	<0.001
Insulin secretagogue subgroup						
Early SMBG	462	9.38	6.75	6.72	6.75	6.77
No early SMBG	2221	9.40	7.29	7.10	7.15	7.15
Difference in HbA1c reduction			-0.52	-0.36	-0.39	-0.36
P-value			<0.001	0.01	0.007	0.01
SMBG	240	9.52	6.71	6.62	6.59	6.66
No SMBG	2033	9.37	7.29	7.11	7.16	7.16
Difference in HbA1c reduction			-0.72	-0.63	-0.72	-0.65
P-value			<0.001	0.002	<0.001	0.001
Non-insulin secretagogue subgroup						
Early SMBG	463	8.06	6.45	6.35	6.37	6.42
No early SMBG	1334	7.54	6.54	6.41	6.43	6.46
Difference in HbA1c reduction			-0.61	-0.58	-0.58	-0.56
P-value			<0.001	<0.001	<0.001	<0.001
SMBG	258	7.92	6.36	6.26	6.27	6.32
No SMBG	1191	7.47	6.54	6.42	6.44	6.47
Difference in HbA1c reduction			-0.63	-0.61	-0.62	-0.60
P-value			<0.001	0.002	<0.001	0.001

Notes. p-value: for interaction effect of SMBG group and time.

Abbreviations: SMBG, self-monitoring of blood glucose; HbA1c, hemoglobin A1c.

Supplementary Table S2

Basic characteristics of participants: insulin secretagogue subgroup (n = 2683).

	SMBG Group (+/+), n=240	SMBG Group (+/-), n=222	SMBG Group (-/+), n=188	SMBG Group (-/-), n=2033	*P-value*
Age at onset (years)	55.0±11.5	54.8±12.2	54.5±11.1	56.6±11.3	0.005
Gender: Male	143 (59.6%)	128 (57.7%)	107 (56.9%)	1061 (52.2%)	0.059
Level of education: No	15 (6.3%)	26 (11.7%)	11 (5.9%)	343 (16.9%)	<0.001
Primary school	64 (26.7%)	69(31.1%)	63 (33.5%)	826 (40.6%)	
High school	111 (46.3%)	89 (40.1%)	84 (44.7%)	667 (32.8%)	
University or above	50 (20.8%)	38 (17.1%)	30 (16.0%)	198(9.7%)	
Family history of DM: Yes	122 (50.8%)	109 (49.1%)	96 (51.1%)	761 (37.4%)	<0.001
Smoking	40 (16.7%)	38(17.1%)	28 (14.9%)	374 (18.4%)	0.61
Alcohol drinking	23 (9.6%)	18 (8.1%)	10 (5.3%)	140 (6.9%)	0.31
Physical activity: No exercise	89(37.2%)	65(34.6%)	107(48.2%)	1309(65.2%)	<0.001
Occasional exercise	54(22.5%)	48(25.5%)	43(19.4%)	244(12.2%)	
Regular exercise	97(40.4%)	75(39.9%)	72(32.4%)	456(22.7%)	
Knowledge regarding GC: Yes	207 (88.8%)	175 (81.8%)	142 (78.0%)	785 (41.8%)	<0.001
Willingness toward DSM: Yes	204 (87.6%)	192 (89.7%)	161(88.5%)	1477 (78.7%)	<0.001
Medication adherence: Yes	228 (97.9%)	209 (97.7%)	176 (96.7%)	1845 (95.7%)	0.21
Clinical variables					
HbA1c at baseline (%)	9.5±2.7	9.2±2.4	9.7±2.4	9.4±2.5	0.20
BMI (kg/m^2)	26.4±4.3	26.7±4.1	26.6±4.2	26.2±4.1	0.25
SBP (mmHg)	127.2±16.3	131.1±17.3	130.1±16.1	133.4±18.8	<0.001
DBP (mmHg)	77.8±9.4	78.7±11.0	80.2±10.6	80.8±11.5	<0.001
Total cholesterol (mg/dL)	170.2±38.9	174.6±37.9	186.0±41.0	195.4±45.0	<0.001
Triglycerides (mg/dL)	148.1±155.4	152.2±104.9	159.0±113.0	174.7±176.4	0.028
HDL-C (mg/dL)	44.5±12.1	45.41±12.5	46.2±11.6	48.3±15.9	<0.001
LDL-C (mg/dL)	99.1±33.0	102.6±30.4	111.8±33.5	115.9±34.7	<0.001
eGFR (mL/min/1.73m^2)	97.4±27.5	96.9±31.4	98.8±34.2	86.0±27.2	<0.001
GPT (U/L)	35.1±39.3	32.5±27.3	35.6±25.2	35.1±31.0	0.67
Comorbidity: CCI	2.1±1.6	1.9±1.3	1.8±1.2	1.7±1.2	<0.001

Notes. Results are expressed as mean±SD or n (%). Abbreviations: SMBG, self-monitoring of blood glucose; SD, standard deviation; DM, diabetes mellitus; GC, glycaemic control; DSM, diabetes self-management; HbA1c, hemoglobin A1c; BMI, body mass index; SBP, systolic blood pressure; DBP, diastolic blood pressure; HDL-C, high-density lipoprotein cholesterol; LDL-C, low-density lipoprotein cholesterol; eGFR, estimated glomerular filtration rate; GPT, glutamic pyruvic transaminase; CCI, Charlson comorbidity index.

Supplementary Table S3

Mean HbA1c levels during the observation period, anti-diabetic medications and main comorbidities in each group: insulin secretagogue subgroup (n = 2683).

	SMBG Group (+/+), n=240	SMBG Group (+/-), n=222	SMBG Group (-/+), n=188	SMBG Group (-/-), n=2033	*P-value*
HbA1c (%)					
at baseline	9.5±2.7	9.2±2.4	9.7±2.4	9.4±2.5	0.20
at 3 months	6.7±1.0	6.6±1.0	7.2±1.4	7.3±1.5	<0.001
at 6 months	6.6±1.0	6.8±1.1	7.0±1.2	7.1±1.3	<0.001
at 9 months	6.5±0.9	6.9±1.1	7.0±1.2	7.2±1.3	<0.001
at 12 months	6.6±0.9	6.9±1.2	7.0±1.3	7.2±1.3	<0.001
Insulin secretagogues					
Sulfonylurea	217 (90.4%)	191 (86.0%)	158 (84.0%)	1677 (82.5%)	0.011
Glinides	39 (16.3%)	49 (22.1%)	48 (25.5%)	514 (25.3%)	0.016
Non-insulin secretagogues					
Metformin	224 (93.3%)	200 (90.1%)	169 (89.9%)	1538 (75.6%)	<0.001
DPP-4 inhibitors	65 (27.1%)	46 (20.7%)	40 (21.3%)	143 (7.0%)	<0.001
Thiazolidinediones	15 (6.3%)	10 (4.5%)	13 (6.9%)	205 (10.1%)	0.009
Acarbose	33 (13.8%)	33 (14.9%)	35 (18.6%)	318 (15.6%)	0.57
Comorbidity					
CHF	29 (12.1%)	28 (12.6%)	17 (9.0%)	183 (9.0%)	0.18
CAD	16 (6.7%)	16 (7.2%)	16 (8.5%)	171 (8.4%)	0.76
CVA	22 (9.2%)	14 (6.3%)	4 (2.1%)	144 (7.1%)	0.031
cancer	11 (4.6%)	4 (1.8%)	3 (1.6%)	14 (0.7%)	<0.001

Notes. Results are expressed as mean±SD or n (%). Abbreviations: SMBG, self-monitoring of blood glucose; SD, standard deviation; HbA1c, hemoglobin A1c; DM, diabetes mellitus; DPP-4, dipeptidyl deptidase 4; CHF, congestive heart failure; CAD, coronary artery disease; CVA, cerebrovascular accident.

Supplementary Table S4

Basic characteristics of participants: non-insulin secretagogue subgroup (n = 1797).

	SMBG Group (+/+), n=258	SMBG Group (+/-), n=205	SMBG Group (-/+), n=143	SMBG Group (-/-), n=1191	*P-value*
Age at onset (years)	54.3±10.8	55.9±11.5	53.5±10.6	56.9±11.6	<0.001
Gender: Male	136 (52.7%)	101 (49.3%)	84 (58.7%)	558 (46.9%)	0.029
Level of education: No	9 (3.5%)	9 (4.4%)	5 (3.5%)	135 (11.3%)	<0.001
Primary school	52 (20.2%)	57 (27.8%)	36 (25.2%)	388 (32.6%)	
High school	116 (45.0%)	86 (42.0%)	59 (41.3%)	456 (38.3%)	
University or above	81 (31.4%)	53 (25.9%)	43 (30.1%)	212 (17.8%)	
Family history of DM: Yes	132 (51.2%)	112 (54.6%)	82 (57.3%)	509 (42.7%)	<0.001
Smoking	30 (11.6%)	24 (11.7%)	23 (16.1%)	154 (12.9%)	0.59
Alcohol drinking	15 (5.8%)	19 (9.3%)	9 (6.3%)	73 (6.1%)	0.38
Physical activity: No exercise	78 (30.4%)	45 (31.5%)	85 (42.3%)	595 (50.6%)	<0.001
Occasional exercise	61 (23.7%)	37 (25.9%)	39 (19.4%)	194 (16.5%)	
Regular exercise	118 (45.9%)	61 (42.7%)	77 (38.3%)	388 (33.0%)	
Knowledge regarding GC: Yes	210 (93.8%)	173 (92.5%)	123 (87.9%)	742 (68.1%)	<0.001
Willingness toward DSM: Yes	189 (84.4%)	168 (89.8%)	124 (88.6%)	904 (82.9%)	0.050
Medication adherence: Yes	215 (96.0%)	177 (95.2%)	135 (96.4%)	1045 (95.3%)	0.90
Clinical variables					
HbA1c at baseline (%)	7.9±1.9	8.3±2.3	8.0±2.0	7.5±1.8	<0.001
BMI (kg/m^2)	26.5±4.6	26.6±4.3	27.1±4.7	27.1±4.3	0.086
SBP (mmHg)	126.1±15.9	126.4±18.5	129.0±17.6	130.8±17.3	<0.001
DBP (mmHg)	78.0±10.1	77.7±12.4	79.4±11.9	79.3±10.6	0.095
Total cholesterol (mg/dL)	170.8±36.8	172.0±36.3	173.0±36.7	179.3±38.3	0.001
Triglycerides (mg/dL)	130.7±133.4	130.8±85.8	132.3±84.9	148.4±117.3	0.027
HDL-C (mg/dL)	45.4±11.1	45.4±10.8	45.2±11.9	47.0±12.3	0.050
LDL-C (mg/dL)	99.4±31.2	100.9±31.4	101.9±29.9	105.0±32.4	0.033
eGFR (mL/min/1.73m^2)	100.8±32.7	103.6±33.2	97.6±25.8	90.4±27.7	<0.001
GPT (U/L)	28.3±16.6	29.6±19.4	35.7±26.1	33.6±25.9	0.001
Comorbidity: CCI	1.9±1.3	1.9±1.4	1.7±1.2	1.9±1.3	0.61

Notes. Results are expressed as mean±SD or n (%). Abbreviations: SMBG, self-monitoring of blood glucose; SD, standard deviation; DM, diabetes mellitus; GC, glycemic control; DSM, diabetes self-management; HbA1c, hemoglobin A1c; BMI, body mass index; SBP, systolic blood pressure; DBP, diastolic blood pressure; HDL-C, high-density lipoprotein cholesterol; LDL-C, low-density lipoprotein cholesterol; eGFR, estimated glomerular filtration rate; GPT, glutamic pyruvic transaminase; CCI, Charlson comorbidity index.

Supplementary Table S5

Mean HbA1c levels during the observation period, anti-diabetic medications, and main comorbidities in each group: non-insulin secretagogue subgroup (n = 1797)

	SMBG Group (+/+), n=258	SMBG Group (+/-), n=205	SMBG Group (-/+), n=143	SMBG Group (-/-), n=1191	*P-value*
HbA1c (%), mean±SD					
at baseline	7.9±1.9	8.3±2.3	8.0±2.0	7.5±1.8	<0.001
at 3 months	6.4±0.7	6.5±0.8	6.4±0.8	6.4±0.9	0.005
at 6 months	6.3±0.6	6.4±0.7	6.2±0.6	6.4±0.7	<0.001
at 9 months	6.3±0.6	6.4±0.7	6.3±0.7	6.4±0.7	0.001
at 12 months	6.3±0.6	6.5±0.8	6.2±0.6	6.5±0.8	<0.001
Insulin secretagogues					
Sulfonylurea	0	0	0	0	--
Glinides	0	0	0	0	--
Non-insulin secretagogues					
Metformin	248 (96.1%)	198 (96.6%)	133 (93.0%)	1069 (89.8%)	<0.001
DPP-4 inhibitors	46 (17.8%)	45 (22.0%)	24 (16.8%)	110 (9.2%)	<0.001
Thiazolidinediones	10 (3.9%)	12 (5.9%)	4 (2.8%)	71 (6.0%)	0.27
Acarbose	38 (14.7%)	23 (11.2%)	23 (16.1%)	178 (15.0%)	0.52
Comorbidity					
CHF	47 (18.2%)	43 (21.0%)	22 (15.4%)	165 (13.9%)	0.034
CAD	15 (5.8%)	10 (4.9%)	11 (7.7%)	105 (8.8%)	0.14
CVA	18 (7.0%)	11 (5.4%)	7 (4.9%)	62 (5.2%)	0.71
cancer	9 (3.5%)	9 (4.4%)	7 (4.9%)	48 (4.0%)	0.91

Notes. Results are expressed as mean±SD or n (%). Abbreviations: SMBG, self-monitoring of blood glucose; SD, standard deviation; HbA1c, hemoglobin A1c; DM, diabetes mellitus; DPP-4, dipeptidyl deptidase 4; CHF, congestive heart failure; CAD, coronary artery disease; CVA, cerebrovascular accident.

Supplementary Table S6

Longitudinal HbA1c trajectory by generalized estimating equations: insulin secretagogue subgroup (n = 2683).

	Early SMBG		1-year SMBG	
	Group (+/+, +/-) vs Group (-/-, -/+)		Group (+/+) vs Group (-/-)	
	**Adjusted β (se)**	**P-value**	**Adjusted β (se)**	**P-value**
**Intercept**	7.10 (0.27)	<0.001	7.44 (0.25)	<0.001
**SMBG group:** No				
Yes	0.06 (0.10)	0.51	0.21 (0.13)	0.13
**Time:** Baseline				
months	-2.12 (0.06)	<0.001	-2.08 (0.06)	<0.001
6 months	-2.30 (0.06)	<0.001	-2.26 (0.06)	<0.001
9 months	-2.25 (0.06)	<0.001	-2.21 (0.06)	<0.001
12 months	-2.25 (0.06)	<0.001	-2.22 (0.06)	<0.001
**Interaction of SMBG group and time**				
Baseline				
3 months	-0.52 (0.14)	<0.001	-0.72 (0.19)	<0.001
6 months	-0.36 (0.14)	0.01	-0.63 (0.20)	0.002
9 months	-0.39 (0.14)	0.007	-0.72 (0.19)	<0.001
12 months	-0.36 (0.14)	0.01	-0.65 (0.19)	0.001
Age at onset (years)	-0.008 (0.002)	<0.001	-0.009 (0.002)	<0.001
Gender: Male	-0.18 (0.04)	<0.001	-0.19 (0.04)	<0.001
Level of education: No				
Primary school	-0.05 (0.06)	0.40	-0.08 (0.07)	0.20
High school	-0.16 (0.07)	0.02	-0.18 (0.08)	0.02
University or above	-0.24 (0.09)	0.004	-0.27 (0.09)	0.004
Smoking	0.16 (0.05)	0.003	0.15 (0.06)	0.01
Alcohol drinking	--	--	-0.14 (0.06)	0.03
Physical activity: No exercise				
Occasional exercise	-0.01 (0.05)	0.88	0.03 (0.06)	0.65
Regular exercise	-0.10 (0.04)	0.01	-0.08 (0.04)	0.046
Medication adherence: Yes	-0.45 (0.11)	<0.001	-0.44 (0.11)	<0.001
HbA1c at baseline (%)	0.28 (0.01)	<0.001	0.29 (0.01)	<0.001
BMI (kg/m^2)	0.011 (0.004)	0.02	--	--
SBP (mmHg)	0.002 (0.001)	0.03	0.003 (0.001)	0.01
Total cholesterol (mg/dL)[a]	0.011 (0.004)	0.006	--	--
Triglycerides (mg/dL)[a]	--	--	0.003 (0.001)	0.04
Charlson comorbidity index	--	--	-0.028 (0.014)	0.049

Notes. Results are expressed as regression coefficients (β) with their corresponding standard error (se). Backward elimination method was adopted to select variables.

[a] per 10 unit

Abbreviations: SMBG, self-monitoring of blood glucose; HbA1c, hemoglobin A1c; BMI, body mass index; SBP, systolic blood pressure.

Supplementary Table S7
Longitudinal HbA1c trajectory by generalized estimating equations: non-insulin secretagogue subgroup (n = 1797).

	Early SMBG		1-year SMBG	
	Group (+/+, +/-) vs Group (-/-, -/+)		Group (+/+) vs Group (-/-)	
	**Adjusted β (se)**	**P-value**	**Adjusted β (se)**	**P-value**
**Intercept**	4.98 (0.17)	<0.001	4.72 (0.15)	<0.001
**SMBG group:** No				
Yes	0.39 (0.08)	<0.001	0.34 (0.09)	<0.001
**Time:** Baseline				
3 months	-1.00 (0.05)	<0.001	-0.93 (0.05)	<0.001
6 months	-1.13 (0.05)	<0.001	-1.05 (0.05)	<0.001
9 months	-1.11 (0.05)	<0.001	-1.03 (0.05)	<0.001
12 months	-1.08 (0.05)	<0.001	-1.00 (0.05)	<0.001
**Interaction of SMBG group and time**				
3 months	-0.61 (0.11)	<0.001	-0.63 (0.13)	<0.001
6 months	-0.58 (0.11)	<0.001	-0.61 (0.13)	<0.001
9 months	-0.58 (0.11)	<0.001	-0.62 (0.13)	<0.001
12 months	-0.56 (0.11)	<0.001	-0.60 (0.13)	<0.001
Gender: Male	-0.07 (0.03)	0.004	-0.07 (0.03)	0.008
Smoking	--	--	0.10 (0.05)	0.04
Physical activity: No exercise				
Occasional exercise	-0.06 (0.03)	0.07	--	--
Regular exercise	-0.06 (0.02)	0.02	--	--
Willingness toward DSM: Yes	-0.07 (0.03)	0.03	--	--
Medication adherence: Yes	-0.16 (0.07)	0.03	--	--
HbA1c at baseline (%)	0.29 (0.01)	<0.001	0.29 (0.01)	<0.001
BMI (kg/m^2)	0.012 (0.003)	<0.001	0.013 (0.003)	<0.001
Total cholesterol (mg/dL)[a]	0.012 (0.004)	0.001	0.013 (0.003)	<0.001
HDL-C (mg/dL)[a]	0.023 (0.011)	0.03	--	--

Notes. Results are expressed as regression coefficients (β) with their corresponding standard error (se). Backward elimination method was adopted to select variables.
[a] per 10 unit
Abbreviations: SMBG, self-monitoring of blood glucose; HbA1c, hemoglobin A1c; DSM, diabetes self-management; BMI, body mass index; HDL-C, high-density lipoprotein cholesterol.

Supplementary Table S8

The association between the frequency of SMBG at baseline and longitudinal HbA1c trajectory models by generalized estimating equations: all participants (n = 4987).

	Adjusted β (se)	P-value
**SMBG group:** No		
< 7 times/week	-0.07 (0.10)	0.48
≥ 7 times/week	1.02 (0.14)	<0.001
**Time:** Baseline		
3 months	-1.69 (0.04)	<0.001
6 months	-1.84 (0.04)	<0.001
9 months	-1.80 (0.04)	<0.001
12 months	-1.79 (0.04)	<0.001
**Interaction of SMBG group and time**		
SMBG < 7 times/week		
3 months	-0.11 (0.10)	0.30
6 months	0.039 (0.11)	0.72
9 months	-0.004 (0.11)	0.97
12 months	0.013 (0.10)	0.90
SMBG ≥7 times/week		
3 months	-1.32 (0.14)	<0.001
6 months	-1.32 (0.14)	<0.001
9 months	-1.30 (0.15)	<0.001
12 months	-1.27 (0.15)	<0.001

Note. The model was adjusted for age at onset, gender, level of education, smoking status, physical activity, medication adherence, HbA1c at baseline, body mass index, systolic blood pressure, triglycerides, high-density lipoprotein cholesterol, low-density lipoprotein cholesterol, using insulin secretagogues, and Charson comorbidity index. Results are expressed as regression coefficients (β) with their corresponding standard error (se).

Abbreviations: SMBG, self-monitoring of blood glucose; HbA1c, hemoglobin A1c.
